# Effect of Cultural Themes on Forming Cotard’s Syndrome: Reporting a Case of Cotard’s Syndrome with Depersonalization and Out of Body Experience Symptoms

**Published:** 2013

**Authors:** Alireza Ghaffari Nejad, Ali Mehdizadeh Zare Anari, Fatemeh Pouya

**Affiliations:** 1 Professor, Department of Psychiatry, Beheshti Hospital, Kerman University of Medical Sciences, Kerman, Iran.; 2 Psychiatrist, Kerman Neuroscience Research Center, Student Counseling Center, Kerman University of Medical Sciences, Kerman, Iran.; 3 Department of Anatomy, School of Medicine, Kerman University of Medical Sciences, Kerman, Iran.

**Keywords:** Cotard’s Syndrome, Cultural Themes, Depersonalization

## Abstract

**Objective:** Cotard’s syndrome is a rare psychiatric syndrome. Its core symptom is nihilistic ideation or delusion.

Case Report: A female patient with Cotard’s syndrome symptoms associated with out of body experience and depersonalization, and complicated grief was referred for evaluation. She believed that she was killed by a creature named "Aal" in the Persian folklore

**Conclusions:** Cultural and superstitious beliefs could affect the forming of the complex constellation of the patient’s symptoms including Cotard’s syndrome symptoms. The resolution of symptoms might be achieved step by step.

**Declaration of interest:** None.

## Introduction

Cotard’s syndrome is a rare psychiatric syndrome with nihilistic ideations or delusions, which has been well-known for more than one hundred years. 

Symptoms of Cotard’s syndrome are variable (1). This syndrome is usually associated with other psychiatric or neurological disorders, but it can exist as an isolated disorder (2). A recent survey showed that 0.11% of neurological and 0.62% of psychiatric cases had Cotard’s syndrome (3). It usually appears in the context of mood disorders or schizophrenia, and sometimes may be the background of self-harming behaviors (4). In this case report, the effect of cultural themes on Cotard’s syndrome symptoms was the matter of concern. 

## Case Report

The patient was a 42 year old woman who believed that she was killed by a ghost. She felt and saw many souls wandering around her and described one ghost as a female ghost with long nails, big eyes, and disheveled hair (Aal). In Persian popular beliefs, “Aal” is a female mythical creature who harms ladies just after delivering a baby. The ghost dissects its victim’s abdomen and eats their liver. The victims become sick later, get worse, and die. The patient reported that she saw Aal for the first time thirteen days after delivering her first son thirteen years ago. At that time she began to see souls, and became paranoid and afraid of death associated with depressed mood. The diagnosis of post-partum depression was made for which she was treated. Four years ago her first son had a motorcycle accident and was in a coma for a few days. She claimed that he died for a short time and the fact that he survived was a miracle. 

Her current symptoms began four weeks prior to her referral to our center. She felt that something strange had happened to her. She felt she is nothing and is dead, as that she has been murdered by a ghost (Aal). Gradually the souls communicated with the patient and she had a clear conversation with them, especially when she was talking about daily events to her dead brother, who passed away recently. She also felt that sometimes things become smaller or bigger than their normal size. She had the same feeling in her upper extremities; she felt they had become so large that they were pressing on her throat, and she had a panic episode. 

On mental examination, she had visual and auditory hallucinations without impaired reality testing. She had other perceptual abnormalities including micropsia, macropsia, depersonalization, and derealization. Her cognitive functioning remained intact. Physical and neurological examinations were unremarkable. Electroencephalogram and neuroimaging were normal. 

For her, 750 mg sodium valproate and 4 mg risperidone were administered. Two weeks after initiating treatment she said: “I am not dead now, but I am in a coma and under special care in ICU”. She was talking as if her body was in a coma confined to bed, and her soul was talking to us. The souls which she was feeling around her were less prominent at this time. Three weeks after the treatment, she said: “I have come out of the coma, but I feel that everything is strange and I am dreaming”. At this point she did not see any souls. She also said: “There is one more thing to do. I should return to my birthplace (in a rural area) where the grave of my brother is, and then I'll take back my soul from him”.

## Discussion

Several explanations have tried to formulate the mechanism of Cotard’s syndrome development. One possibility is that there is a neuropsychological anomaly in patients with Cortard’s syndrome. There is more general reduction of affective response to stimuli in patients with Cortard’s syndrome (5).

Young has suggested that an unusual perception could result in Capgras syndrome. 

If the patient uses an externalizing attributional style, Capgras syndrome can develop. If an internalizing attributional style is used, the result will be Cotard’s syndrome (6). However, the concept that Cotard’s and Capgras syndromes are different in attributional style cannot be completely generalized; there are cases of co-occurrence of Cotard’s syndrome with Capgras syndrome (7, 8).

That Cotard’s syndrome starts with an unusual perception was stressed in this theory. In our case, the starting point was with an unusual experience in the form of out of body experience followed with other symptoms. 

As far as we know, there is no previous report of such an experience with Cotard’s syndrome. This patient’s sense of depersonalization and further derealization are two other perceptual abnormalities which are less noticed in the literature. The patient may simultaneously feel that he/she is dead.

Yamada et al. believed that Cotard’s syndrome develops through three stages of germination, blooming, and chronic phases (9). Although we cannot delineate precise phases in our patient’s course, there were distinct phases in her recovery. First she believed she was dead. Next, she was convinced that she was in a coma and near death. Finally, she believed that she was getting out of coma, but the world was very strange. We concluded that Cotard’s syndrome patients might recover gradually, stage by stage, just as it may develop step by step. 

We think our patient felt guilty regarding her brother’s recent death and her son’s head injury. She may have suffered from a form of prolonged and complicated grief, which made her prone to felling guilty. She was afraid of death and maybe she used Cotard’s syndrome as a counterphobic mechanism; there is no fear of death when you have already passed away. It is suggested that regular visual perceptions could change after death, when the body is buried and the soul wonders here and there. Therefore, micropsia, macropsia, and strange feelings of the world might be understandable in this patient.

The number “thirteen” is an unlucky number in many cultures and in the Persian culture. Many bad events might happen in the first 13-day period of the Persian New Year. Our patient believed that she had seen "Aal" thirteen days after delivering her second son. This child is the same one who had a head injury four years ago. The patient believed that he died and revived later. She described the story as if she and her son are immortal.

Previous episodes of Cotard's syndrome, as she could remember, were the same as her recent episodes (more details could not be obtained). There are many points which should be clarified in Cotard’s syndrome. One of these issues is the influence of cultural beliefs on forming symptom constellation, which we tried to deal with in this report.
